# Successful treatment of acute left main coronary artery disease with a drug-coated balloon under left ventricular unloading using Impella: a case report

**DOI:** 10.1093/ehjcr/ytae443

**Published:** 2024-08-22

**Authors:** Kazuhiro Kamada, Kensuke Joko, Naoya Otaka, Hidenori Matsusaka, Kunio Morishige

**Affiliations:** Department of Cardiovascular Medicine, Matsuyama Red Cross Hospital, 1 Bunkyocho, Matsuyama-city, Ehime 790-8524, Japan; Department of Cardiovascular Medicine, Matsuyama Red Cross Hospital, 1 Bunkyocho, Matsuyama-city, Ehime 790-8524, Japan; Department of Cardiovascular Medicine, Matsuyama Red Cross Hospital, 1 Bunkyocho, Matsuyama-city, Ehime 790-8524, Japan; Department of Cardiovascular Medicine, Matsuyama Red Cross Hospital, 1 Bunkyocho, Matsuyama-city, Ehime 790-8524, Japan; Department of Cardiovascular Medicine, Matsuyama Red Cross Hospital, 1 Bunkyocho, Matsuyama-city, Ehime 790-8524, Japan

**Keywords:** Drug-coated balloon, Stentless percutaneous coronary intervention, Mechanical haemodynamic support, Impella, Left ventricular unloading, Left main coronary artery disease, Case report

## Abstract

**Background:**

Although the efficacy and safety of drug-coated balloons (DCBs) for acute left main coronary artery (LMCA) disease have not yet been proven, stentless percutaneous coronary intervention with a DCB is preferred for patients with high bleeding risk requiring a shorter duration of dual antiplatelet therapy. Mechanical circulatory support may improve haemodynamics in patients with cardiogenic shock caused by acute LMCA disease.

**Case summary:**

A 74-year-old man diagnosed with acute congestive heart failure underwent emergency coronary angiography (CAG) at our hospital owing to ischaemic changes on the electrocardiogram (ECG), indicating acute LMCA disease. Coronary angiography revealed severe LMCA ostial stenosis. Immediately after CAG, mechanical circulatory support was initiated using Impella CP® for haemodynamic collapse with abrupt ST-segment elevation in the precordial leads. The haemodynamics stabilized with a dramatic improvement in the ECG. We treated the culprit ostial lesion with inflation of a cutting balloon followed by DCB delivery because of an episode of haematochezia. Subsequently, his cardiac function recovered fully.

**Discussion:**

A case of acute LMCA disease was successfully treated with a DCB under haemodynamic support using Impella CP. The left ventricular (LV) unloading with Impella was indicated to contribute to stable haemodynamics, even during long inflation with the DCB, and the immediate recovery of LV function. Haemodynamic support using Impella may be effective, especially in cases requiring repeated and longer inflation of balloon catheters accompanied by extensive myocardial ischaemia.

**Learning points**

- Drug-coated balloons (DCBs) might be preferred in patients with high bleeding risk requiring a shorter duration of dual antiplatelet therapy.
- Left ventricular (LV) unloading with Impella CP® could stabilize haemodynamics and enable long inflation with a DCB for left main coronary artery (LMCA) disease.
- Immediate LV unloading with Impella (or a percutaneous LV assist device) could contribute to reducing the infarct size and LV function recovery in acute LMCA disease.

## Introduction

Stentless percutaneous coronary intervention (PCI) with drug-coated balloons (DCBs) is effective and safe in treating *de novo* lesions in small vessels and in-stent restenosis.^1^ Although the use of DCB for *de novo* lesions in large vessels [reference vessel diameter (RVD) ≧ 3.0 mm] is allowed, there are few reports regarding its efficacy and safety compared with drug-eluting stents (DES). Additionally, the efficacy and safety of stentless PCI with DCB for ST-segment elevation myocardial infarction (STEMI) involving left main coronary artery (LMCA) disease remains unknown.

Impella® (axial flow pump, ABIOMED, MA, USA) is one of the most commonly used device in cardiogenic shock.^2^ Impella could reduce the left ventricular pressure (LVP) and LV end-diastolic volume (LVEDV) and stabilize haemodynamics.^3^

We present a case of acute heart failure (HF) followed by cardiogenic shock owing to acute LMCA disease, successfully treated by stentless PCI with a DCB under hemodynamic support with Impella CP®.

## Summary figure

**Table:** 

Day 1	Admission with acute congestive HF due to acute LMCA disease. After tracheal intubation, PCI with a DCB was performed under haemodynamic support with the Impella CP.
Day 3	Successful removal of the Impella CP.
Day 4	Successful weaning from the ventilator.
Day 9	Follow-up CAG revealed no restenosis.
Day 11	Transthoracic echocardiography showed almost normal LV ejection fraction.
Day 15	Colonoscopy revealed rectal cancer.
Day 17	Discharged.

## Case presentation

A 74-year-old man presented to the emergency department with acute dyspnoea. He had a present diagnosis of hypertension and type 2 diabetes mellitus and had experienced one episode of haematochezia in the past year, but did not consult a doctor. On examination, his blood pressure was 108/81 mmHg and his heart rate was 107 b.p.m. His pulse oxygen saturation level was 91% with oxygen inhalation (10 L/min on a reservoir mask). Physical examination revealed no heart murmurs and peripheral oedema, but pulmonary crackles. Laboratory results showed decreased haemoglobin [10.8 g/dL (normal range, 13.7–16.8 g/dL)], normal creatine kinase (CK), myocardial component of CK (CKMB) [126 U/L (59–248 U/L) and 4 U/L (≤5 U/L), respectively], normal troponin I [35.6 pg/mL (≤47.0 pg/mL)], elevated lactate dehydrogenase [10.5 mmol/L (0.4–1.8 mmol/L)], and elevated brain natriuretic peptide [347.8 pg/mL (≤18.4 pg/mL)] levels.

**Figure 1:**
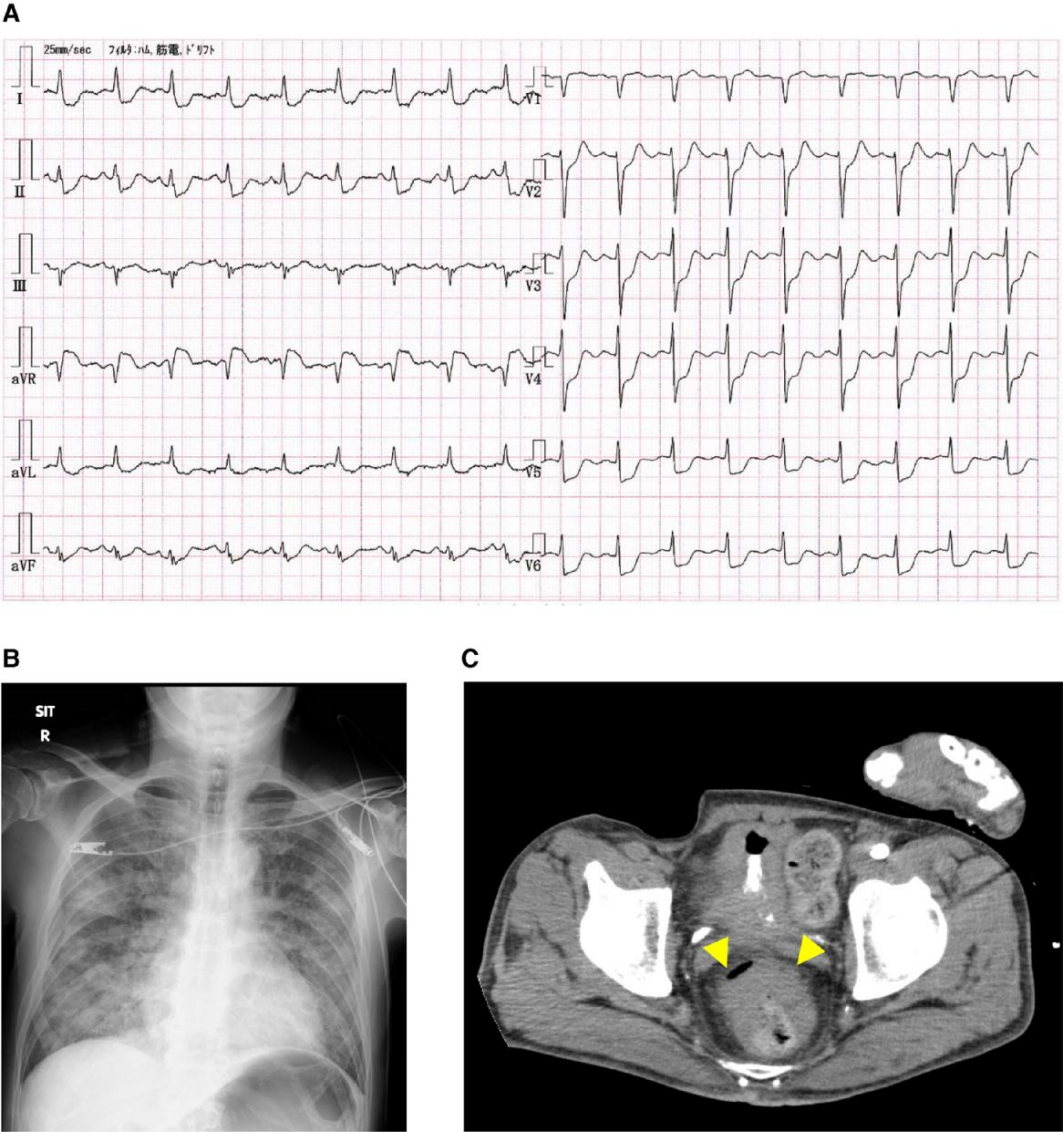
(*A*) The electrocardiogram shows sinus tachycardia and ST-segment depression in all leads except for aVR, which showed ST-segment elevation. (*B*) Chest radiography shows mild cardiomegaly (cardiothoracic ratio 52%) and pulmonary congestion. (*C*) Plain abdominal computed tomography revealed the thickening of the rectal wall (arrow heads).

A 12-lead electrocardiogram (ECG) showed sinus tachycardia and ST-segment depression in all leads except the aVR which showed ST-segment elevation (*Figure 1A*). Chest radiography revealed mild cardiomegaly and pulmonary congestion (*Figure 1B*). Transthoracic echocardiography (TTE) showed mildly reduced LV ejection fraction (LVEF) of 40%–50% with diffuse LV wall motion hypokinesis but no local asynergy, and no significant valvular disease. Plain abdominal computed tomography revealed rectal wall thickening, suggesting rectal cancer (*Figure 1C*).

**Figure 2:**
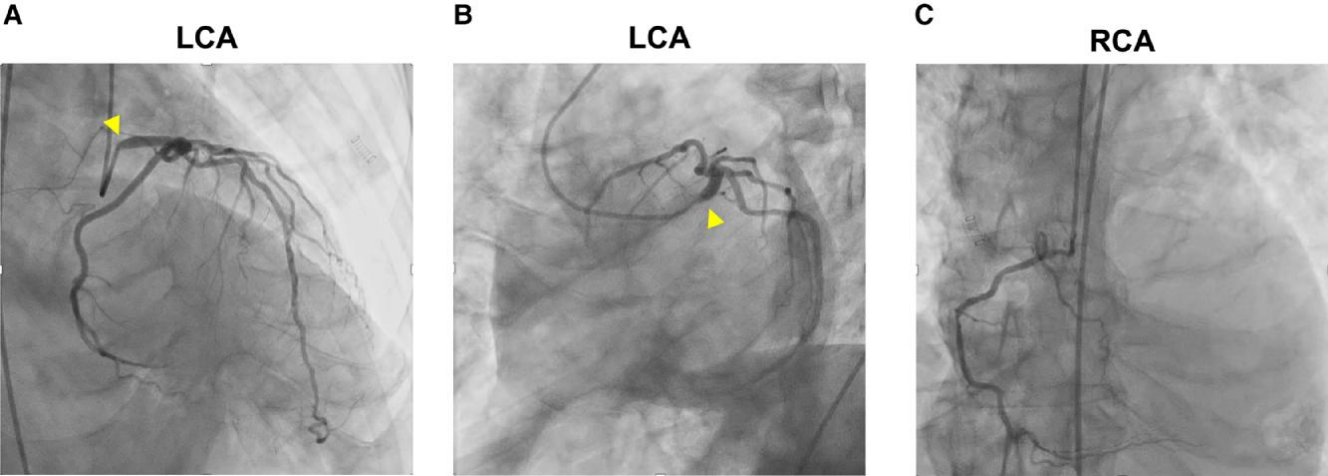
Emergent coronary angiography. (*A*) Left main coronary artery ostium shows severe stenosis (arrow heads; right anterior oblique cranial view). (*B*) LMCA culprit lesion (arrow heads; left anterior oblique caudal view). (*C*) A hypoplastic right coronary artery.

**Figure 3:**
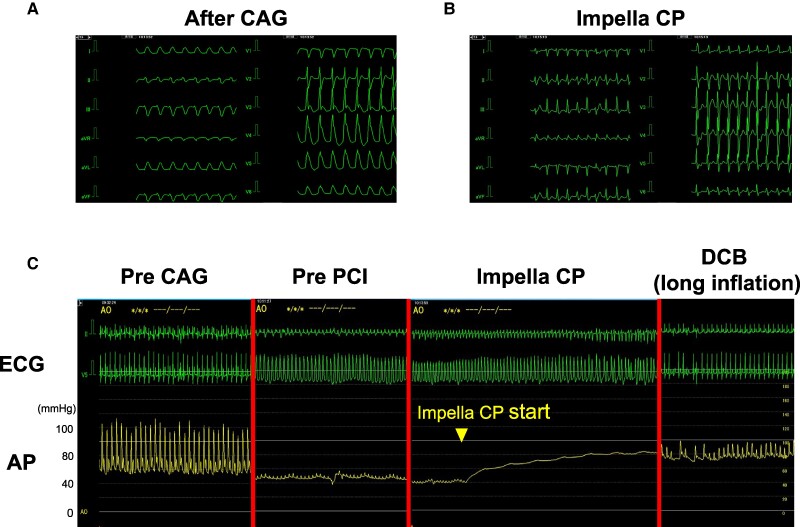
(*A*) The electrocardiogram shows ST-segment elevation in Ⅰ, aVL, and V3–6 leads after coronary angiography. (*B*) ST-segment elevation is immediately improved after initiating hemodynamic support with Impella CP. (*C*) The left panel shows the electrocardiogram and arterial pressure pre–coronary angiography. After coronary angiography, the mean arterial pressure decreased to around 40 mmHg with electrocardiogram changes (second panel from the left). Once initiating haemodynamic support with Impella CP, the arterial pressure immediately increased to ∼80 mmHg (second panel from the right). During long inflation of the drug-coated balloon for left main coronary artery disease, the haemodynamics remained stable under haemodynamic support with Impella CP (right panel).

**Figure 4:**
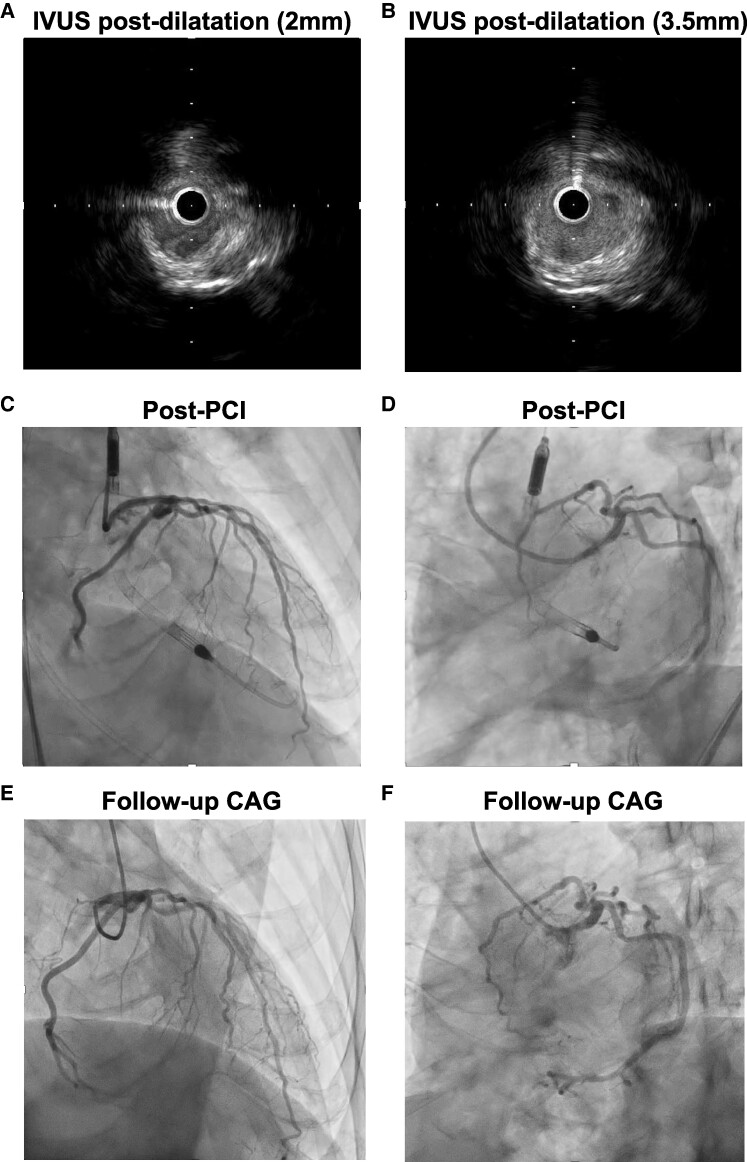
(*A*) Intravascular ultrasound after pre-dilatation with a non-compliant balloon reveals the calcified plaque (minimum lumen area of 3.1 mm^2^). (*B*) IVUS after pre-dilatation with a cutting balloon reveals satisfactory dilatation (MLA of 6.1 mm^2^). (*C*) Coronary angiography post–percutaneous coronary intervention shows a satisfactory dilatation result (right anterior oblique cranial view). (*D*) Coronary angiography post–percutaneous coronary intervention (left anterior oblique caudal view). (*E*) Follow-up coronary angiography on hospital day 9 revealed no restenosis (right anterior oblique cranial view). (*F*) Follow-up coronary angiography on hospital day 9 (left anterior oblique caudal view).

**Figure 5:**
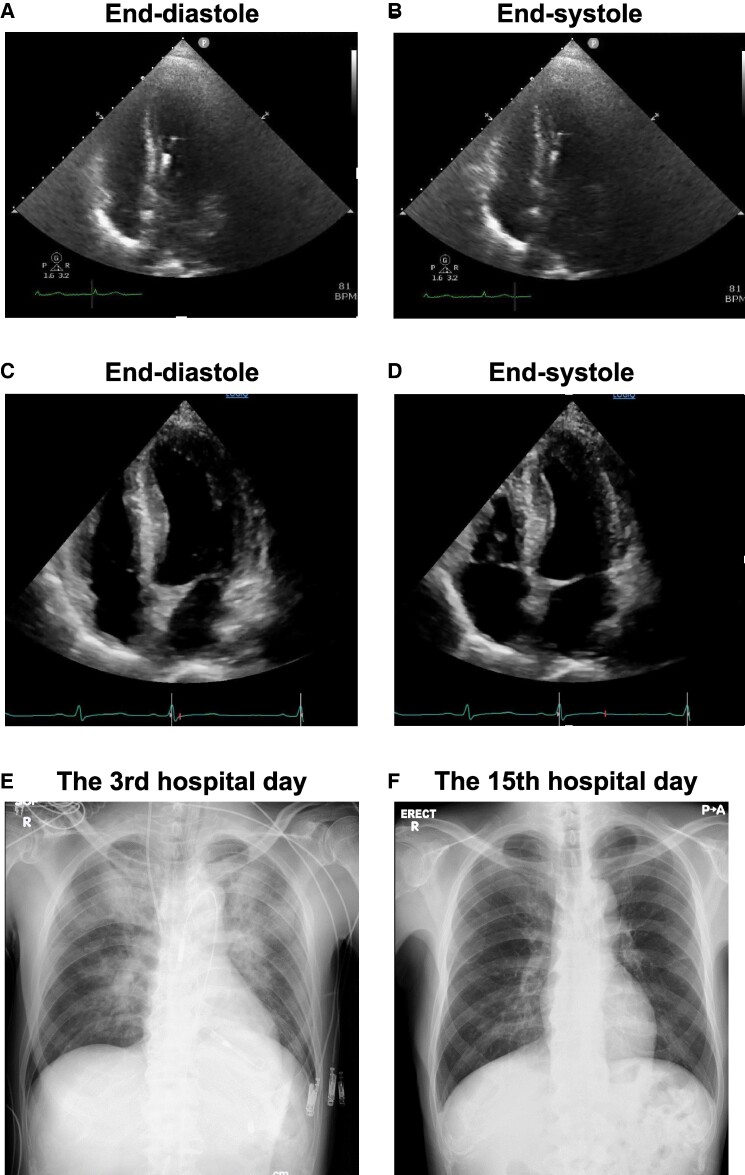
(*A* and *B*) Transthoracic echocardiography showing low left ventricular ejection fraction (38%) on hospital day 2. (*C* and *D*) Transthoracic echocardiography showing normal left ventricular ejection fraction (57%) on hospital day 11. (*E*) Chest radiography after Impella CP on hospital day 3. (*F*) Chest radiography showing improvement in cardiomegaly and pulmonary congestion on hospital day 15.

The patient was diagnosed with acute congestive HF, possibly caused by extensive myocardial ischaemia. First, we planned to treat the HF with positive pressure ventilation using adaptive servo-ventilation (ASV) and medication. The patient’s symptoms improved immediately after wearing the ASV; however, 6 h later, he required invasive mechanical ventilation under tracheal intubation for acute worsening of oxygenation. Emergency coronary angiography (CAG) revealed severe stenosis in the LMCA ostium (*Figure 2A* and *B*) and a hypoplastic right coronary artery (*Figure 2C*). While preparing for PCI, the ECG showed ST-segment elevation in leads I, aVL, and V3–6, and his mean arterial pressure (MAP) decreased to ∼40 mmHg (*Figure 3A*). We assumed that coronary occlusion by a 5  Fr catheter (Judkins Left) could disrupt haemodynamics because the catheter can easily wedge into the LMCA ostium during CAG. We immediately inserted the Impella CP via a left femoral approach to provide haemodynamic support; his MAP subsequently increased to ∼80 mmHg and ECG changes improved (*Figure 3B* and *C*). Percutaneous coronary intervention was performed under stable haemodynamics to the LMCA ostium after an aspirin (200 mg) and a prasugrel (20 mg) were administered as a loading dose of dual antiplatelet therapy (DAPT). We placed a 6 Fr guide catheter (ASAHI Hyperion JL4.0STSH, ASAHI INTECC, Japan) in the LMCA ostium and delivered a guidewire (ASAHI SION, ASHI INTECC, Japan) to the left anterior descending artery. After pre-dilatation with a non-compliant balloon (Hiryu Plus 2.0 × 15 mm, TERUMO, Japan), we evaluated the LMCA using intravascular ultrasound (IVUS; Alta View, TERUMO, Japan), which revealed a calcified plaque (∼300°) and RVD of 4.5 mm and minimum lumen area (MLA) of 3.1 mm^2^ (*Figure 4A*; Supplementary material online, *Video S1*). We inflated a cutting balloon (WOLVERINE 3.5 × 10 mm, Boston Scientific Corporation, MA, USA) at the rated burst pressure in the culprit lesion for 30 s. Subsequent angiography showed 30% residual stenosis, and IVUS confirmed sufficient vessel dilatation without flow-limiting dissection (MLA 6.1 mm^2^; *Figure 4B–D*; Supplementary material online, *Video S2*). A shorter duration of DAPT is preferable for rectal malignancies. Thus, we performed stentless PCI with a paclitaxel-coated DCB (SeQuent Please NEO 3.5 × 20 mm, NIPRO, Japan), and prolonged inflation for 2 min was possible without worsening of haemodynamics under the support of Impella CP (*Figure 3C*). The peak CK and CKMB levels were 3640 U/L and 130 U/L, respectively, 6 h after PCI. Although LVEF was temporarily reduced immediately after PCI (*Figure 5A* and *B*; Supplementary material online, *Video S3*), it rapidly improved to near-normal levels. Since we confirmed the stable haemodynamics without catecholamine, we removed the Impella CP 2 days after PCI. Moreover, the patient was weaned from the ventilator on hospital day 4. On hospital day 9, right heart catheterization showed 3.1 L/min/m^2^ of cardiac index and 1 mmHg of mean pulmonary capillary wedge pressure, corresponding to Forrester Ⅰ. A follow-up CAG revealed no restenosis of the treated lesions (*Figure 4E* and *F*). Transthoracic echocardiography showed normal LVEF (57%) with no local asynergy (*Figure 5C* and *D*; Supplementary material online, *Video S4*). Chest radiography revealed improvements in cardiomegaly and pulmonary congestion (*Figure 5E* and *F*). Colonoscopy detected rectal cancer requiring further investigation. The patient was discharged on day 17 of hospitalization without complications. Aspirin (100 mg) and prasugrel (3.75 mg) were continued as maintenance doses of DAPT for a month followed by prasugrel (3.75 mg) monotherapy; the approved maintenance dose of prasugrel is 3.75 mg daily in Japan.

## Discussion

To date, many studies have reported the superiority or non-inferiority of DCB compared to DES for major adverse cardiovascular events (MACEs) and target lesion revascularization in *de novo* lesions in small vessels.^1^ In the SPARTAN-LMS study, comparing the all-cause and cardiac mortality of patients treated with DCB to those with DES for *de novo* LMCA lesion, there was no significant difference between the two groups with a median follow-up of 33 months. Additionally, the DCB group was treated with a significantly shorter duration of DAPT.^4^

In the STEMI setting, the REVELATION trial found that DCB was non-inferior to DES in terms of the mean fractional flow reserve value at 9 months after emergent PCI.^5^ Although there have been no studies regarding the effectiveness of DCB for acute LMCA disease, stentless PCI with DCB could be beneficial for LMCA disease, considering the aforementioned evidence.

Although we used a cutting balloon for lesion modification, rotational atherectomy (RA) and intravascular lithotripsy (IVL) have been reported to lead to successful lesion modification in patients with calcified coronary lesions compared with modified balloons.^6,7^ Therefore, the use of RA or IVL needs to be considered.

We used a paclitaxel-coated balloon, which had shown significantly less angiographic late lumen loss at 6 months compared to a sirolimus-coated balloon.^8^ Generally, DCB requires an inflation time > 30 s to provide a sufficient amount of paclitaxel to the vessel wall. In addition to the setting of acute LMCA disease, longer inflation of balloon catheters could worsen haemodynamics. In high-risk PCI involving LMCA disease, there are many studies regarding the role of mechanical haemodynamic support, such as intra-aortic balloon pump (IABP) and Impella. Perera D *et al*. reported that prophylactic IABP insertion before high-risk PCI showed a significant survival advantage in terms of 5-year all-cause mortality compared to the no-planned IABP groups.^9^ The USpella/cVAD registry documented that the early initiation of Impella prior to high-risk PCI was feasible and safe.^10^ Additionally, in the DanGer Shock trial, the routine use of Impella CP under STEMI-related cardiogenic shock reduced the risk of death from any cause at 180 days than standard care alone.^11^ Moreover, Impella CP can reduce the LVP and LVEDV, leading to a reduction in the pressure–volume area, that is, LV unloading. Left ventricle unloading could reduce the infarct size proportionate to the degree of LV unloading.^3^ In our case, when support with Impella CP was initiated, the pulse pressure was almost flat, indicating that the total cardiac output almost depended on Impella CP. We believe that Impella is effective, especially in extensive myocardial ischaemia, because the degree of LV unloading becomes higher upon the dependence on Impella. For percutaneous mechanical circulatory support, we can consider IABP and extracorporeal membrane oxygenation (ECMO). However, both IABP-only support and ECMO combined with IABP do not lead to a reduction in the pressure–volume area.^12,13^

In patients with high bleeding risk (HBR), a shorter duration of DAPT after PCI is favoured. In the MASTER DAPT trial, net adverse clinical events and MACE were comparable between the abbreviated DAPT therapy (1 month) and standard DAPT therapy (DAPT ≧ 3 months) in patients undergoing PCI with DES. Additionally, bleeding events were significantly reduced in the abbreviated DAPT therapy.^14^ Therefore, we continued DAPT for a month followed by prasugrel monotherapy.

We report a case of acute LMCA disease successfully treated with a DCB under LV unloading with Impella CP. Stentless PCI with a DCB was preferred considering HBR and localization of the culprit lesion in our case. Impella CP markedly stabilized the haemodynamics and enabled revascularization with a DCB for the LMCA ostium. Furthermore, LV function dramatically recovered after the treatment. We believe that LV unloading by Impella is highly effective, especially in extensive myocardial ischaemia due to acute LMCA disease. We expect that the DCB would be effective and safe in acute LMCA disease.

## Lead author biography


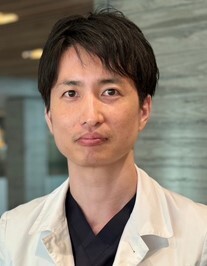


Kazuhiro Kamada works as a physician and cardiology fellow at Matsuyama Red Cross Hospital, Japan. He graduated with his PhD degree from Kyushu University Graduate School of Medical Sciences. He is interested in the field of catheter intervention.

## Supplementary Material

ytae443_Supplementary_Data

## Data Availability

The data underlying this article are available in the article and its online Supplementary material.
